# Supplementary material for: Proximal and distal effects of genetic susceptibility to multiple sclerosis on the T cell epigenome
Source: Nat Commun. 2021 Dec 6;12:7078. doi: 10.1038/s41467-021-27427-w (PMC8648735; doi:10.1038/s41467-021-27427-w)
Supplement: Supplementary file 1 — Reporting Summary [file 41467_2021_27427_MOESM1_ESM.pdf]

nature research

Corresponding author(s): Philip L. De Jager, MD, PhD

Last updated by author(s): Nov 6, 2021

Reporting Summary

Nature Research wishes to improve the reproducibility of the work that we publish. This form provides structure for consistency and transparency in reporting. For further information on Nature Research policies, see our Editorial Policies and the Editorial Policy Checklist.

Statistics

For all statistical analyses, confirm that the following items are present in the figure legend, table legend, main text, or Methods section.

n/a	Confirmed	
☐	☒	The exact sample size (*n*) for each experimental group/condition, given as a discrete number and unit of measurement
☐	☒	A statement on whether measurements were taken from distinct samples or whether the same sample was measured repeatedly
☐	☒	The statistical test(s) used AND whether they are one- or two-sided<br>*Only common tests should be described solely by name; describe more complex techniques in the Methods section.*
☐	☒	A description of all covariates tested
☐	☒	A description of any assumptions or corrections, such as tests of normality and adjustment for multiple comparisons
☐	☒	A full description of the statistical parameters including central tendency (e.g. means) or other basic estimates (e.g. regression coefficient) AND variation (e.g. standard deviation) or associated estimates of uncertainty (e.g. confidence intervals)
☐	☒	For null hypothesis testing, the test statistic (e.g. *F*, *t*, *r*) with confidence intervals, effect sizes, degrees of freedom and *P* value noted<br>*Give P values as exact values whenever suitable.*
☐	☒	For Bayesian analysis, information on the choice of priors and Markov chain Monte Carlo settings
☒	☐	For hierarchical and complex designs, identification of the appropriate level for tests and full reporting of outcomes
☐	☒	Estimates of effect sizes (e.g. Cohen's *d*, Pearson's *r*), indicating how they were calculated

Our web collection on statistics for biologists contains articles on many of the points above.

Software and code

Policy information about availability of computer code

Data collection	DNA methylation beta scores were derived using Illumina's GenomeStudio software v2011.1.
Data analysis	Bioconductor packages minfi v1.26.0 and wateRmelon v1.22.0 were used for the preprocessing of DNA methylation data. PLINK 2.0 was used for the preprocessing of genetic data. Whole-genome imputation of the genetic data was performed using the Michigan Imputation Server. HLA imputation was performed using SNP2HLA v1.0.3. cis- and trans- mQTL analyses were performed using QTLtools v1.1. R coloc package v3.1 was used for colocalization analyses. GLINT v1.0.3 was used for the methylome-wide association studies. FUMA v1.3.3d was used for gene-set enrichment analysis. STAR v2.6.0a, RNA-SeQC v1.1.9 and RSEM v1.3.0 were used for the preprocessing of RNA sequencing data. R v3.4.0 was used for the statistical analyses. R mediation package v4.4.6 was used for mediation analyses.

For manuscripts utilizing custom algorithms or software that are central to the research but not yet described in published literature, software must be made available to editors and reviewers. We strongly encourage code deposition in a community repository (e.g. GitHub). See the Nature Research guidelines for submitting code & software for further information.

Data

Policy information about availability of data

All manuscripts must include a data availability statement. This statement should provide the following information, where applicable:

- Accession codes, unique identifiers, or web links for publicly available datasets
- A list of figures that have associated raw data
- A description of any restrictions on data availability

The genotyping and DNA methylation data from our MS CD4+ T cell dataset have been deposited in the Synapse database under accession codes syn26340457 [https://doi.org/10.7303/syn26340457] and syn26339303 [https://doi.org/10.7303/syn26339303], respectively. As sensitive human data, these data can be accessed upon request, following the establishment of a Data Use Agreement with the Brigham and Women’s Hospital.

The complete set of summary statistics from the cis-mQTL analysis are available for download from the Synapse database under accession code syn26339302 [https://doi.org/10.7303/syn26339302].
The genome-wide significant cis-mQTL effects can be found in Supplementary Data 1.
Data from the Genetics of Lipid-lowering Drugs and Diet Network (GOLDN) study used for the cis-mQTL replication analyses can be obtained from dbGaP under accession code phs000741.v2.p1[https://www.ncbi.nlm.nih.gov/projects/gap/cgi-bin/study.cgi?study_id=phs000741.v2.p1].
BLUEPRINT Epigenome Project's publicly available cis-mQTL summary statistics were downloaded from ftp://ftp.ebi.ac.uk/pub/databases/blueprint/blueprint_Epivar/. BLUEPRINT genetic data were accessed from the European Genome-phenome Archive under accession code EGAD00001002663 [https://ega-archive.org/datasets/EGAD00001002663].

Field-specific reporting

Please select the one below that is the best fit for your research. If you are not sure, read the appropriate sections before making your selection.

- [x] Life sciences
- [ ] Behavioural & social sciences
- [ ] Ecological, evolutionary & environmental sciences

For a reference copy of the document with all sections, see nature.com/documents/nr-reporting-summary-flat.pdf

Life sciences study design

All studies must disclose on these points even when the disclosure is negative.

Sample size	The sample size was not predetermined. Given the sample size of 150-200 participants, SNPs with minor allele count <15 were excluded from the study, so that association analyses are performed with at least 15 samples present in the smaller genotype group. Therefore, in this study, we have effectively assessed the effects of SNPs with minor allele frequency >0.1 on DNA methylation levels.
Data exclusions	Data were excluded for participants with missing genotype data, in addition to samples with the following pre-established criteria: samples from individuals of non-European ancestry and ethnic outliers (>3 standard deviation difference from the European samples on multidimensional scaling of the genetic data), and outlier DNA methylation samples (>3 standard deviation on the first 4 PCs).
Replication	Replication analyses were limited to CpGs measured in the independent datasets: data from BLUEPRINT and GOLDN studies were used for the replication of cis-mQTL findings (95% and 94% of the shared associations showed a similar direction of effect between our study and the BLUEPRINT and GOLDN studies, respectively). Data from BLUEPRINT study were used for the replication of cis-colocalization analyses (of the 8 shared colocalized MS-cis-mQTL effects, 7 were replicated using BLUEPRINT data). Data from BLUEPRINT, UC Berkeley, and PhenoGenetic studies were using to replicate trans-mQTL findings (the effect was replicated in all 3 replication datasets). Data from BLUEPRINT study were used to replicate the cis-methylation effects of MS MHC polygenic score (all shared data were replicated). Data from PhenoGenetic study was used for the replication of trans-methylation effect of MS MHC polygenic score (successful replication). Trans-methylation effect of MS total polygenic score could not be replicated, as the as the target CpG was not available in the 450k methylation array used in the BLUEPRINT study, and the EpiTYPER measurements from the PhenoGenetic study did not pass quality control (40% of the samples did not have a reliable measurement).
Randomization	Randomization is not relevant, as the study is an observational study.
Blinding	Methylation data collection was based on treatment group (blood samples from patients treated with GA or DMF were selected for the study). However, the treatment group is not related to the current study aim (assessing the effects of genetics on DNA methylation). As such, blinding at data collection in not relevant. Data analysis was performed on the hundreds of thousands of methylation sites and genotypes simultaneously in one analysis, without any prior hypotheses per methylation site. Hence, blinding is also not relevant to the data analysis.

Reporting for specific materials, systems and methods

We require information from authors about some types of materials, experimental systems and methods used in many studies. Here, indicate whether each material, system or method listed is relevant to your study. If you are not sure if a list item applies to your research, read the appropriate section before selecting a response.

Materials & experimental systems

n/a	Involved in the study	
[ ]	[x]	Antibodies
[x]	[ ]	Eukaryotic cell lines
[x]	[ ]	Palaeontology and archaeology
[x]	[ ]	Animals and other organisms
[ ]	[x]	Human research participants
[x]	[ ]	Clinical data
[x]	[ ]	Dual use research of concern

Methods

n/a	Involved in the study	
[x]	[ ]	ChIP-seq
[x]	[ ]	Flow cytometry
[x]	[ ]	MRI-based neuroimaging

Antibodies

Antibodies used	T cell activation: anti-human CD3 (BioXCell, Cat# BE0001-2, Clone: OKT-3), anti-human CD28 (BioXCell, Cat# BE0248, Clone: 9.3)

	Flow cytometry: FITC anti-human CD3 (BD Pharmingen, Cat# 555339, Clone: HIT3a), PE anti-human CD4 (BioLegend, Cat# 317409, Clone: OKT4), APC anti-human CD45RA (BD Pharmingen, Cat# 550855, Clone: HI100).
Validation	Technical data and application references can be found at:<br>https://bxcell.com/product/h-cd3/<br>https://bxcell.com/product/h-cd28/<br>https://www.bdbiosciences.com/en-us/products/reagents/flow-cytometry-reagents/research-reagents/single-color-antibodies-ruo/fitc-mouse-anti-human-cd3.555339<br>https://www.biolegend.com/en-us/products/pe-anti-human-cd4-antibody-3654<br>https://www.bdbiosciences.com/en-in/products/reagents/flow-cytometry-reagents/research-reagents/single-color-antibodies-ruo/apc-mouse-anti-human-cd45ra.550855

Human research participants

Policy information about studies involving human research participants

Population characteristics	Subjects were participants in the Comprehensive Longitudinal Investigation of Multiple Sclerosis at the Brigham and Women's Hospital (CLIMB) study. CLIMB is a natural history observational study of MS, in which participants undergo semi-annual neurological examinations and annual magnetic resonance imaging and blood draw, from which peripheral blood mononuclear cells (PBMC) are cryopreserved. In 2015, cryopreserved PBMC samples from subjects meeting the following criteria were pulled from the archive for sample processing: (1) age 18-55 years old, (2) a diagnosis of MS fulfilling 2010 McDonald criteria, (3) a relapsing-remitting disease course at the time of sampling, (4) being on disease-modifying therapies (either glatiramer acetate [GA] or dimethyl fumarate [DMF]) at the time of sampling, (5) no evidence of disease activity in the prior 6 months, (6) no steroid use in the preceding 30 days, and (7) an Expanded Disability Status Scale (EDSS) score between 0 to 4 at the time of sampling. Final samples used in the analysis were from 106 female and 50 male participants, with an average age of 41.6 years old (SD=6.9).
Recruitment	Cryopreserved PBMC samples were accessed from a pre-existing bank collected from participants in the Comprehensive Longitudinal Investigation of Multiple Sclerosis at the Brigham and Women's Hospital (CLIMB) study. Samples were selected based on pre-established inclusion criteria, minimizing selection bias on the results.
Ethics oversight	Institutional Review Board of the Brigham and Women's Hospital.

Note that full information on the approval of the study protocol must also be provided in the manuscript.
