# Supplementary material for: Proximal and distal effects of genetic susceptibility to multiple sclerosis on the T cell epigenome
Source: Nat Commun. 2021 Dec 6;12:7078. doi: 10.1038/s41467-021-27427-w (PMC8648735; doi:10.1038/s41467-021-27427-w)
Supplement: Supplementary file 2 — Supplementary Information [file 41467_2021_27427_MOESM2_ESM.pdf]

Supplementary Information

Proximal and distal effects of genetic susceptibility to multiple sclerosis on the T cell epigenome

Tina Roostaei[1], Hans-Ulrich Klein[1], Yiyi Ma[1], Daniel Felsky[2], Pia Kivisäkk[3], Sarah M. Connor[1], Alexandra Kroshilina[1], Christina Yung[1], Belinda J. Kaskow[4], Xiaorong Shao[5], Brooke Rhead[5], José M. Ordovás[6], Devin M. Absher[7], Donna K. Arnett[8], Jia Liu[9], Nikolaos Patsopoulos[4], Lisa F. Barcellos[5], Howard L. Weiner[4], Philip L. De Jager[1]*

* Correspondence: Philip L. De Jager, MD PhD
pld2115@cumc.columbia.edu

Supplementary Figures 1-11

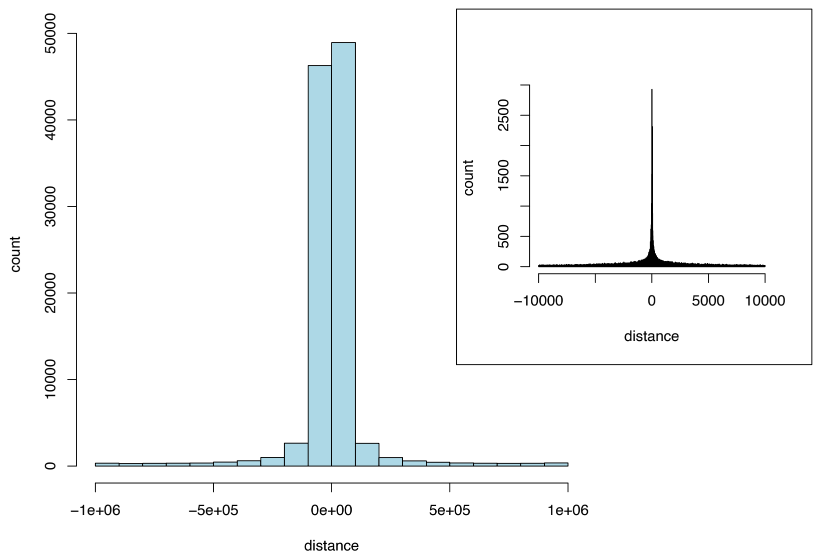

Supplementary Figure 1. Distribution of distance between mSNPs and their target *cis*-mCpGs. Distance distribution is shown in base pairs (1Mb window). Inset: higher resolution illustration in ±10kb.

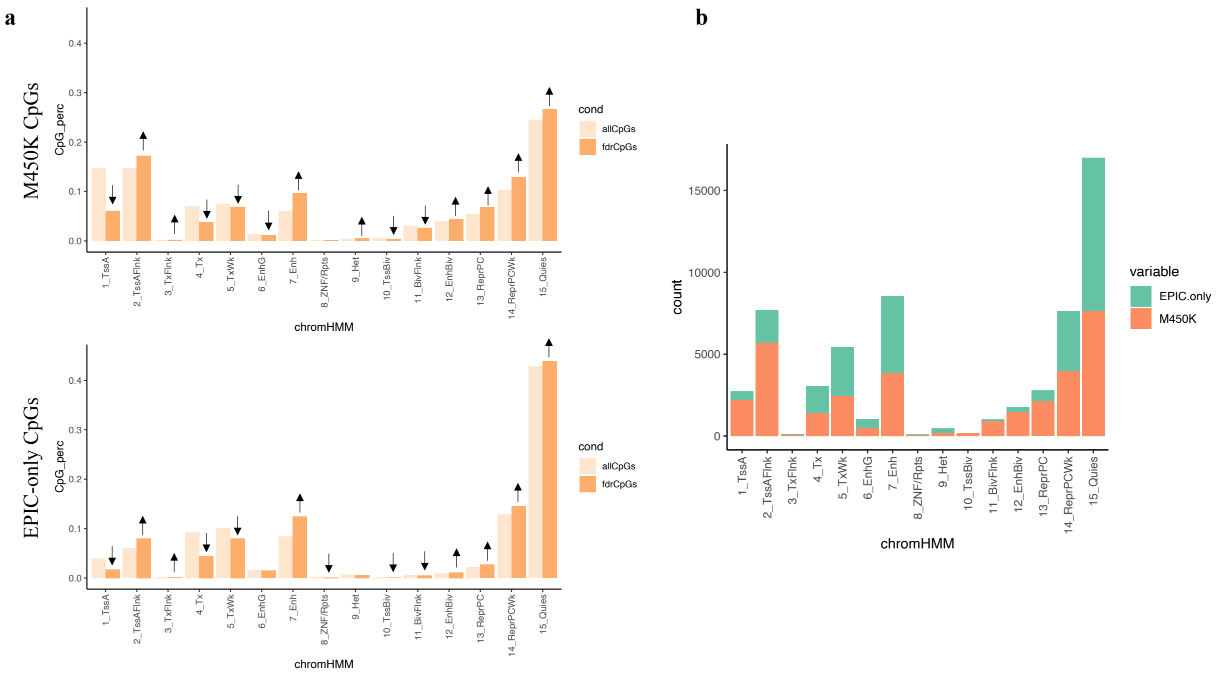

Supplementary Figure 2. Effect of methylation array on the functional distribution of *cis*-mCpGs. (a) Distribution of the identified *cis*-mCpGs in comparison to all tested CpGs in relation to chromatin states modeled for CD4+ T cells (annotations from Roadmap Epigenomics Project, sample #E043) depending on whether the CpGs are available in the Illumina 450K array (top panel, n=52,688) or are only measured in the MethylationEPIC array (bottom panel, n=55,234). Significant enrichment/depletion are shown using upwards/downwards arrows, respectively. **(b)** Frequency of unique functional chromatin segments containing mCpGs (n= 59,603) and their coverage with Illumina 450K vs. MethylationEPIC array.

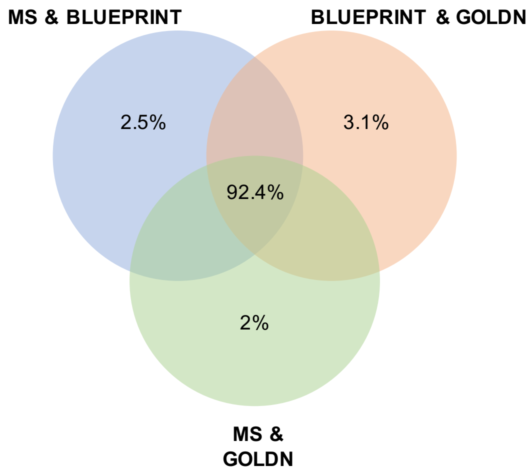

Supplementary Figure 3. Percentage of mQTL effects from our study that show the same direction of effect in BLUEPRINT and GOLDN studies. Data is on the 18,221 mCpG-SNP pairs available from all 3 studies.

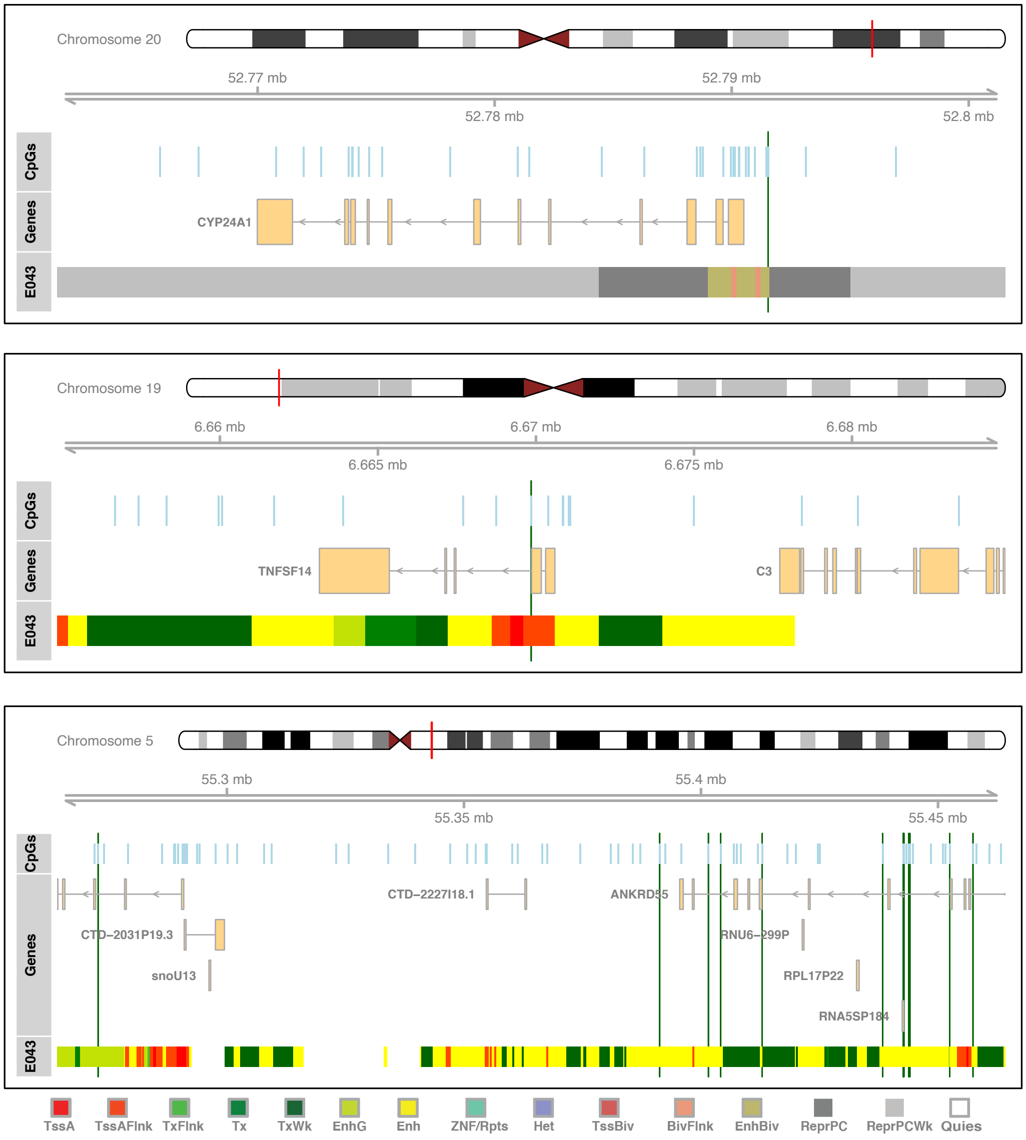

Supplementary Figure 4. Genomic location of mCpGs in the top 3 colocalized MS-*cis*-mQTL loci (depicted in higher resolution). mCpGs with colocalization posterior probability >0.8 in the top 3 colocalized MS-*cis*-mQTL effects illustrated in **Figure 2** are represented with green vertical lines. All CpGs assessed with the Infinium MethylationEPIC kit are shown in light blue. Gene exon/intron positions are based on Ensembl 93. Chromatin state annotations for CD4^{+} T cells are from the Roadmap Epigenomics Project (sample #E043). Genomic positions are in GRCh37 (hg19) coordinates.

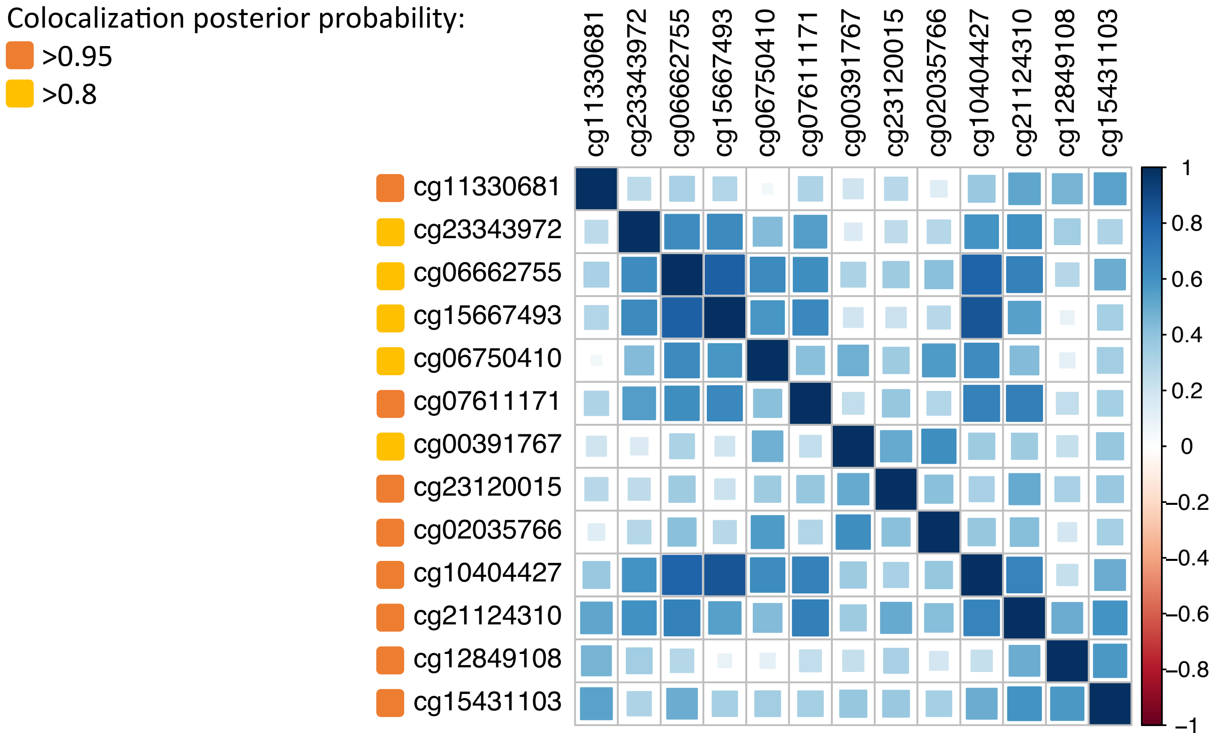

Supplementary Figure 5. Correlation between methylation levels of CpGs affected by rs7731626 MS susceptibility locus. Methylation levels of all the 13 colocalized mCpGs are positively correlated.

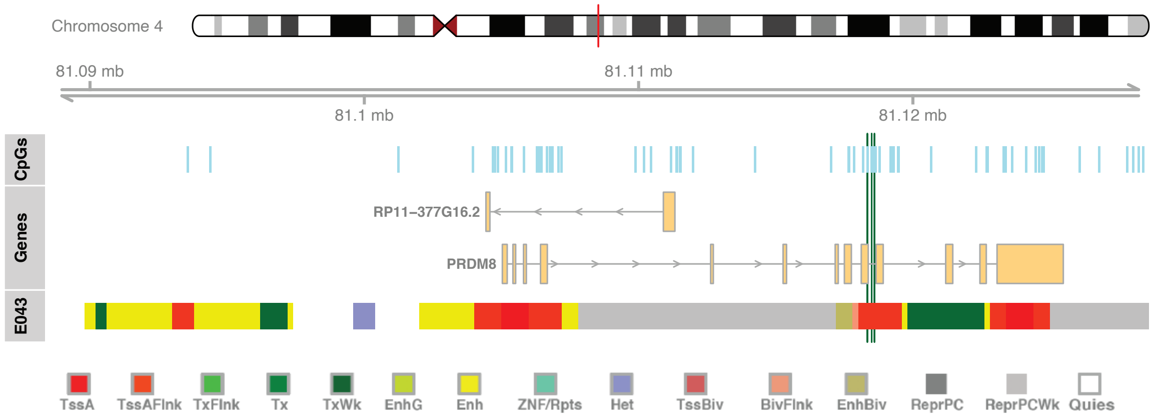

Supplementary Figure 6. Genomic location of mCpGs for the colocalized MS-*trans*-mQTL locus (depicted in higher resolution). mCpGs with colocalization posterior probability >0.95 in the colocalized MS-*trans*-mQTL effect illustrated in **Figure 3** are represented with green vertical lines. All CpGs assessed with the Infinium MethylationEPIC kit are shown in light blue. Gene exon/intron positions are based on Ensembl 93. Chromatin state annotations for CD4^{+} T cells are from the Roadmap Epigenomics Project (sample #E043). Genomic positions are in GRCh37 (hg19) coordinates.

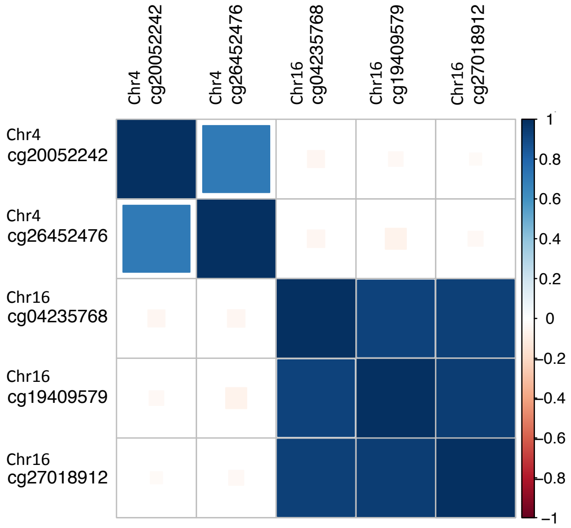

Supplementary Figure 7. Correlation between *cis* and *trans* mCpGs affected by rs3809627 MS locus. No correlation is observed between methylation levels of the 2 *cis*-mCpGs on chromosome 16 and 3 *trans*-mCpGs on chromosome 4.

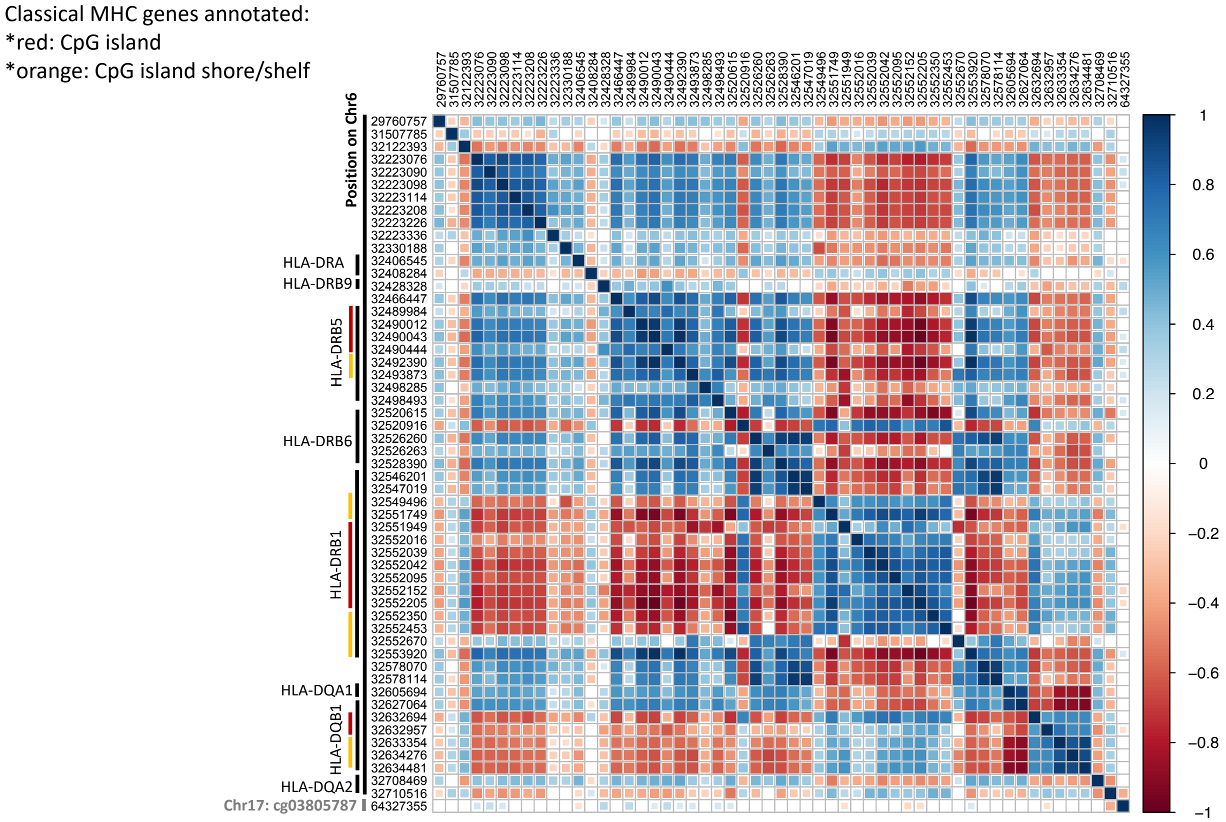

Supplementary Figure 8. Correlation between methylation levels of CpGs affected by MS MHC polygenic score. Genomic coordinates are shown in GRCh37/hg19. All except one CpG are located in chromosome 6. The last CpG is located in chromosome 17.

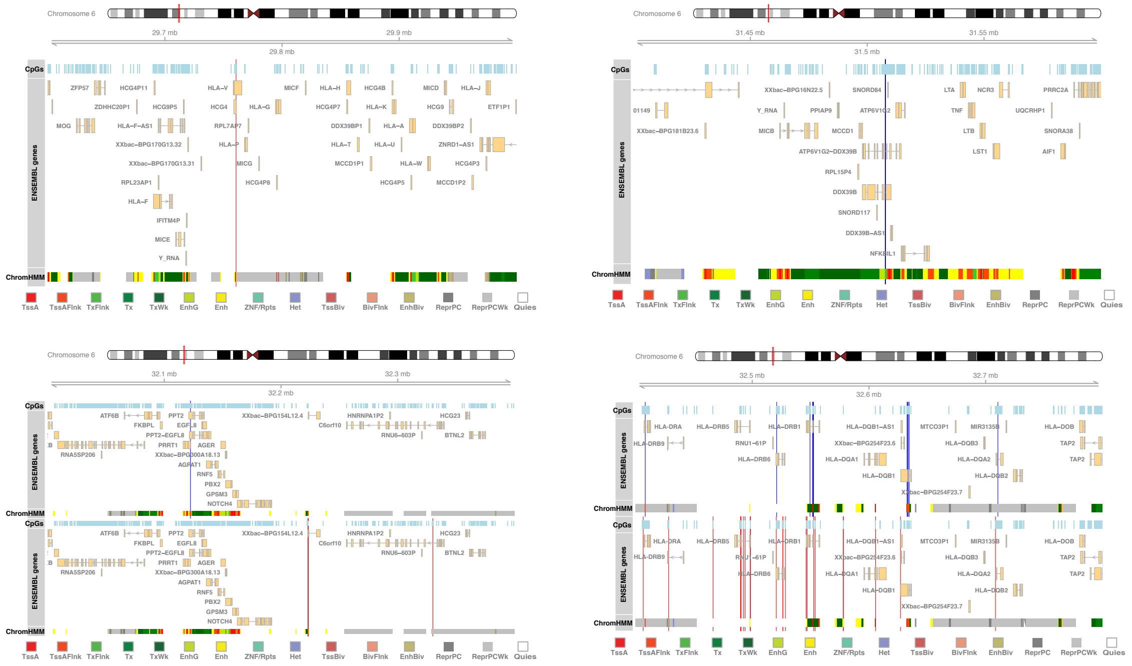

Supplementary Figure 9. Genomic locations of MHC CpGs affected by MS MHC polygenic score in relation to nearby CpGs, genes, and modeled chromatin states. Blue/red vertical lines represent the locations for CpGs with negative/positive associations with the MHC polygenic score, respectively.

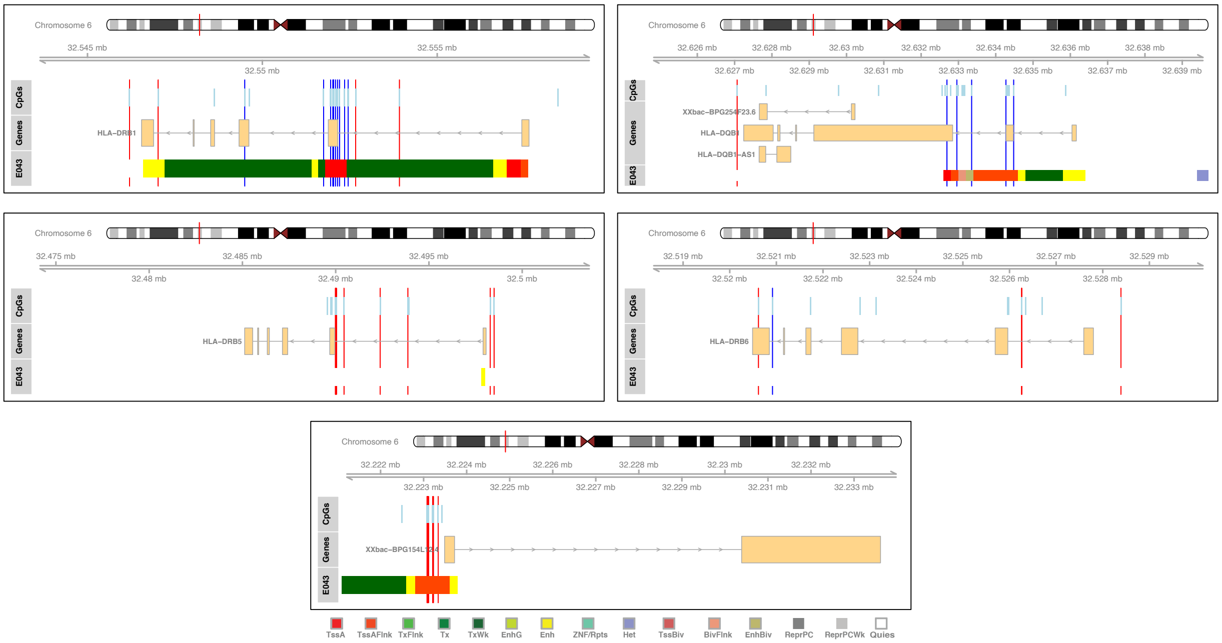

Supplementary Figure 10. Genomic location of CpGs associated with MS MHC polygenic score in genes with several significant hypo- or hyper- methylated CpGs (depicted in higher resolution). Hypo-methylated CpGs are represented with blue and hyper-methylated CpGs are represented with red vertical lines. All CpGs assessed with the Infinium MethylationEPIC kit are shown in light blue. Gene exon/intron positions are based on Ensembl 93. Chromatin state annotations for CD4⁺ T cells are from the Roadmap Epigenomics Project (sample #E043). Genomic positions are in GRCh37 (hg19) coordinates.

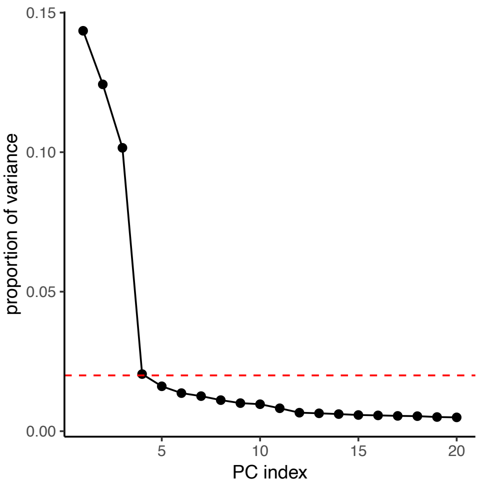

Supplementary Figure 11. Variance explained by methylation PCs. Percentage of variance explained by the first 20 principal components of the methylation data is illustrated. Red dashed line marks 2%.
