# Supplementary material for: Proximal and distal effects of genetic susceptibility to multiple sclerosis on the T cell epigenome
Source: Nat Commun. 2021 Dec 6;12:7078. doi: 10.1038/s41467-021-27427-w (PMC8648735; doi:10.1038/s41467-021-27427-w)
Supplement: Supplementary file 3 — Description of Additional Supplementary Files [file 41467_2021_27427_MOESM3_ESM.pdf]

Description of Additional Supplementary Files

File Name: Supplementary Data 1
Description: Genome-wide significant cis-mQTL effects.

File Name: Supplementary Data 2
Description: Colocalized MS-mQTL effects.

File Name: Supplementary Data 3
Description: . cis-eQTL effects associated with colocalized MS-cismQTL effects.

File Name: Supplementary Data 4
Description: CpGs affected by MS MHC polygenic score (linear regression modeling t-statistics; two-sided P-values corrected for multiple comparisons).

File Name: Supplementary Data 5
Description: CpGs affected by MS total polygenic score (linear regression modeling t-statistics; two-sided P-values corrected for multiple comparisons).

File Name: Supplementary Data 6
Description: MS susceptibility loci associated with cg16050799 methylation (linear regression modeling t-statistics; two-sided nominal Pvalues).

File Name: Supplementary Data 7
Description: MS polygenic score calculation.

File Name: Supplementary Data 8
Description: EpiTYPER analysis primers.
